# Chest wall reconstruction after sternectomy with preservation of sternoclavicular joint function

**DOI:** 10.1016/j.xjtc.2023.09.013

**Published:** 2023-09-16

**Authors:** Rabin Gerrah

**Affiliations:** Department of Cardiothoracic Surgery, Stanford University Cardiovascular Institute, Stanford University, Stanford, Calif

**Figure:**
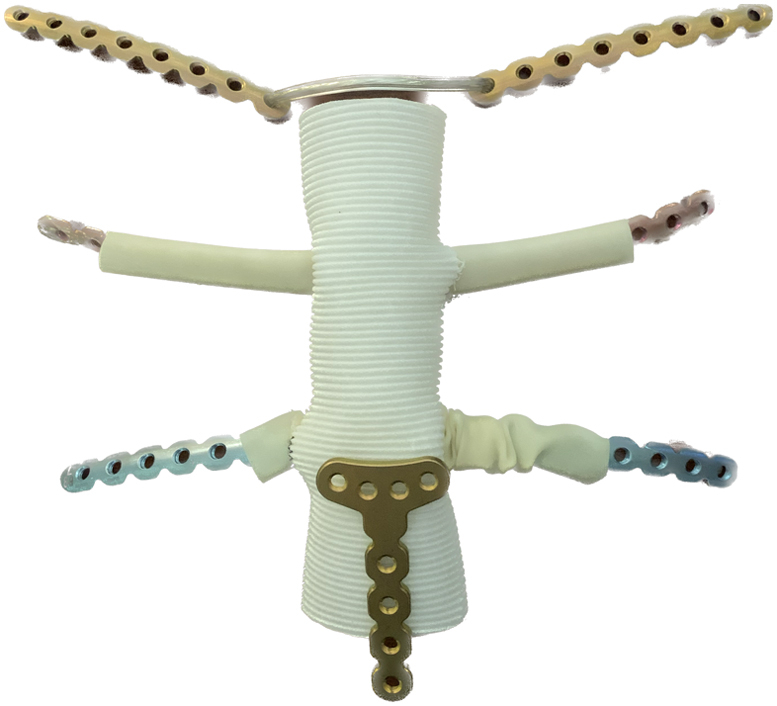
Customized prosthesis to reconstruct the defect after sternectomy and clavicle resection.

**Central Message**

A simple and customizable chest wall reconstruction after sternectomy and medial clavicle resection is presented for complete restoration of native anatomy and the sternoclavicular junction.

Resection of large sternal tumors involving the sternoclavicular joint (SCJ) pose specific reconstruction challenges. The sternum constitutes the point of trust for the ribs and SCJ is the main skeletal connection between the axial skeleton and the upper extremity. Most reconstructive procedures aim for restoration with a rigid structure, stabilizing the resected ribs, covering the mediastinum, and preserving the chest wall integrity. More often, the clavicle stumps are excluded from the reconstruction plan, leaving them free-floating due to the complexity of reconstruction after medial clavicle resection.^1^ The technique described here is a comprehensive method of reconstruction that also preserves SCJ function.

## Technique

**Figure 1:**
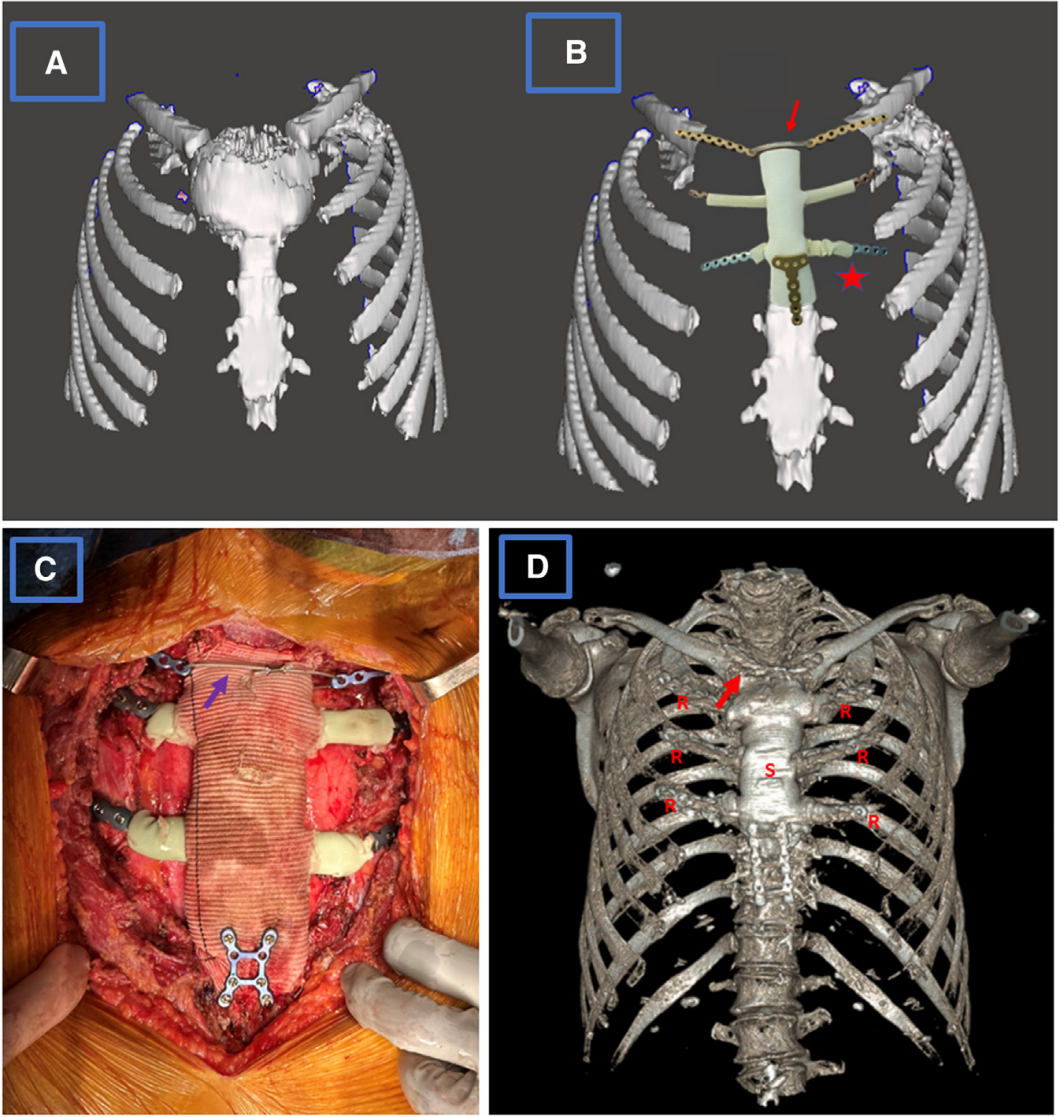
A, Three-dimensional image of the sternal tumor involving the sternoclavicular junction. B, Schematic diagram of the superimposed prosthesis image in the chest wall defect after wide resection of the sternal chondrosarcoma. The *asterisk* shows the crinkled latex tube before fixation of the plate to the rib, after which they are pulled over the ribs and filled with cement. The *arrow* shows the cable connecting the clavicle plates, allowing limited multiaxial hinge motion to replace the sternoclavicular joint. C, Operative view of the reconstruction of sternum, 2 ribs, and sternoclavicular joint with cable connecting the clavicular plates (*arrow*). D, Postoperative scan of the reconstruction of sternum (*S*), 3 ribs (*R*), and sternoclavicular joint with cable (*arrow*).

The patients provided informed consent for the publication of their data. Institutional review board approval was not required for this report. The resection of the tumor is completed by excision of the involved sternum, manubrium, and SCJ with appropriate margins and this includes the medial aspects of clavicles, involved ribs, and soft tissues (Figure 1, *A*). The reconstruction is done by creating a neosternum from bone cement, titanium plates to extend the ribs, and incorporation in the neosternum and cables to attach the clavicles to the neosternum while preserving free motion and mimicking the native SCJ function (Figure 1, *B*).

Initially, 2 sternal wires are placed through the sternal stump as a frame and twisted to add strength in the cast.^2^ A 34-mm polytetrafluoroethylene (PTFE) Hemashield platinum vascular graft (Getinge AB), used as a molding template for the sternum, is placed on the lower sternal stump and affixed to the sternum with sutures. Side-holes (1.5 cm) corresponding to the trajectory of the resected rib stumps are made on each side of the graft. Rib plates with appropriate length, conformed to chest geometry, are placed inside the half-inch diameter latex tube and passed through the graft transversely. While the latex tube is temporarily crinkled and kept medially, the rib plates were affixed with screws to the ribs. The latex tubes are then cut such that they cover 2 cm of the plated rib stump and temporarily tied with a silk suture.

A short plate is affixed to each side of the clavicular stump and a sternal cable (Zimmer Biomet) is passed through the graft, anchored to the twisted wires, and then through the free holes of the clavicular plates.

**Figure E1:**
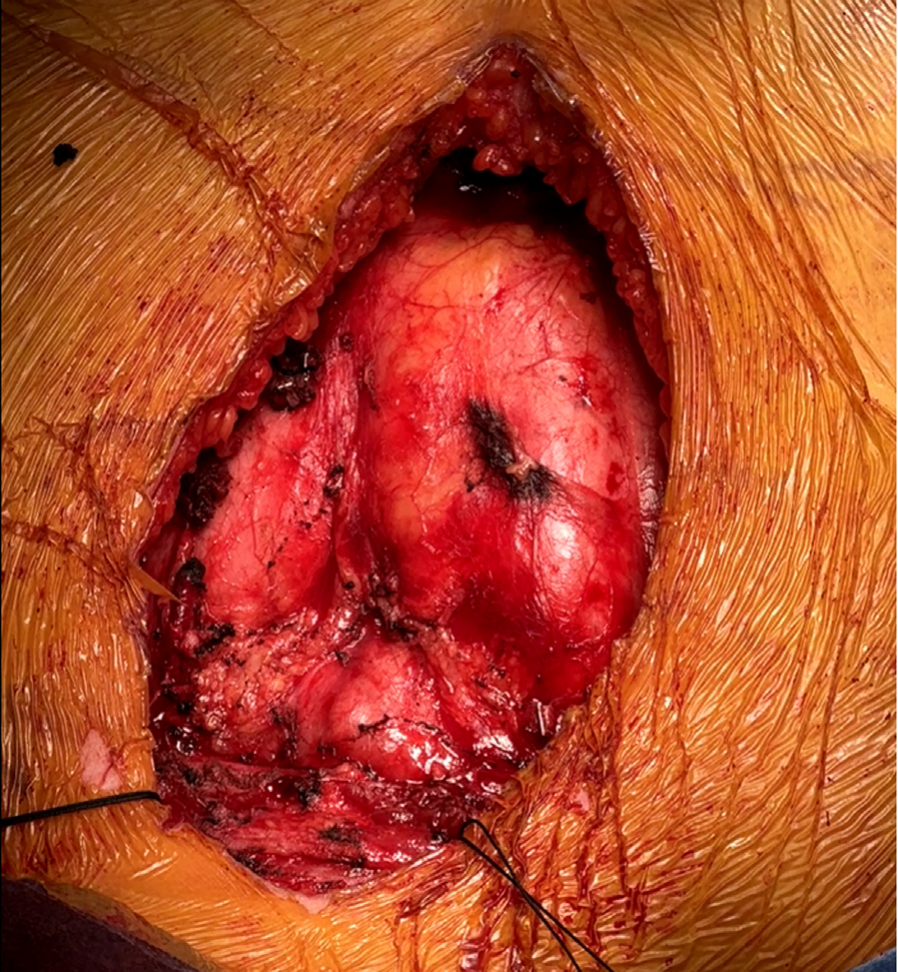
Chest wall defect after resection of the sternum, medial ribs, and medial clavicles.

**Figure E2:**
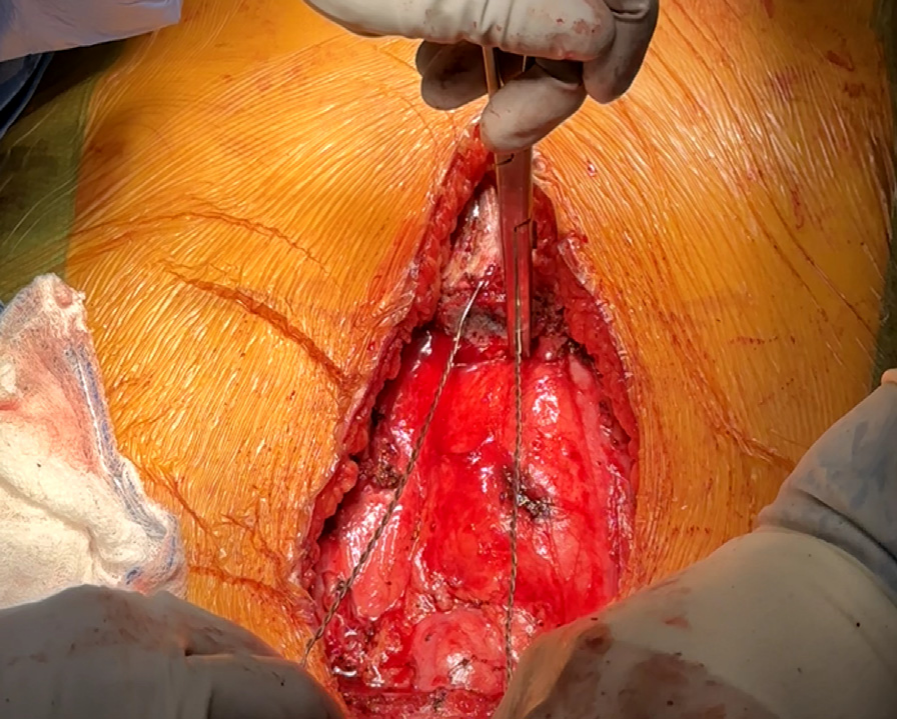
Placement of the twisted sternal wires in the sternal stump.

**Figure E3:**
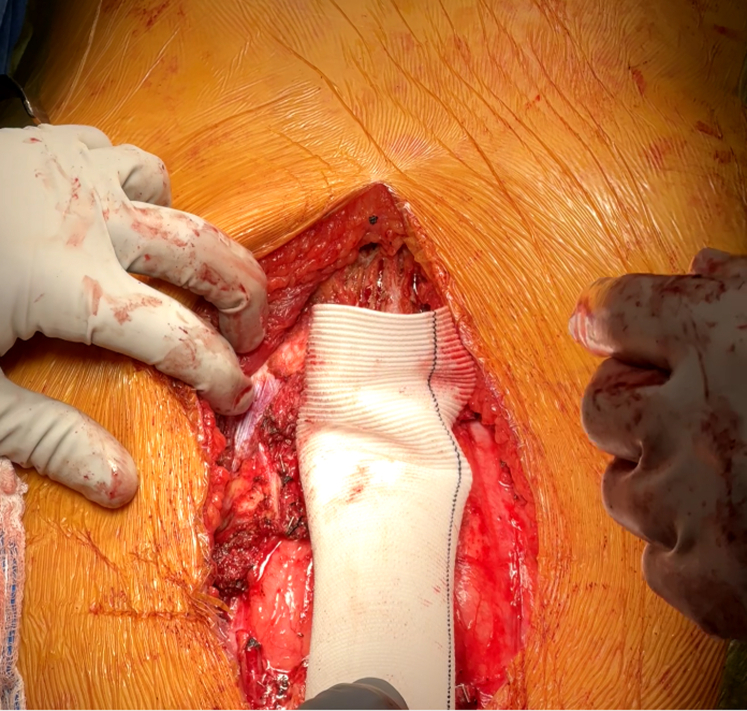
Placement of the Hemashield graft (Getinge AB) over the wires on the sternal stump.

**Figure E4:**
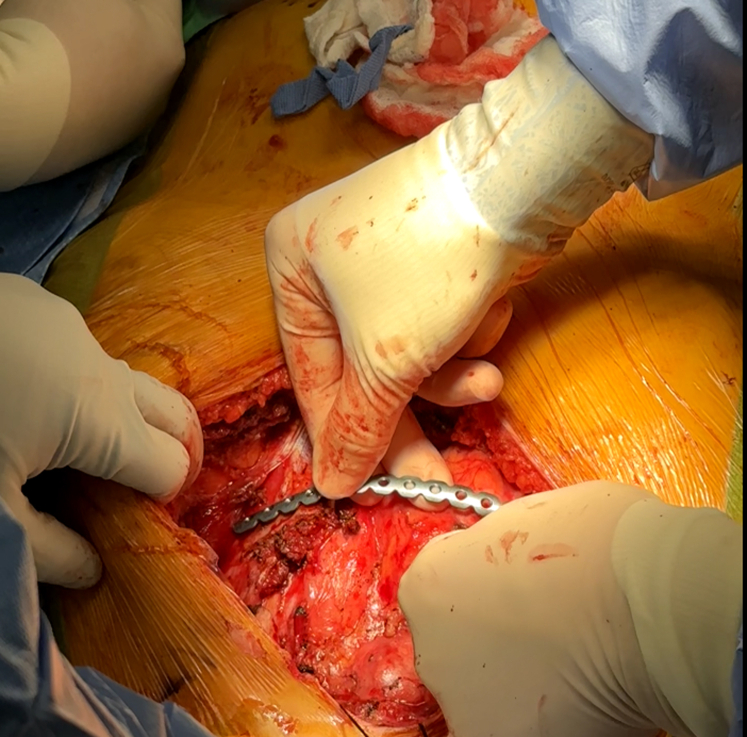
Conforming the plates to appropriate curvature and length.

**Figure E5:**
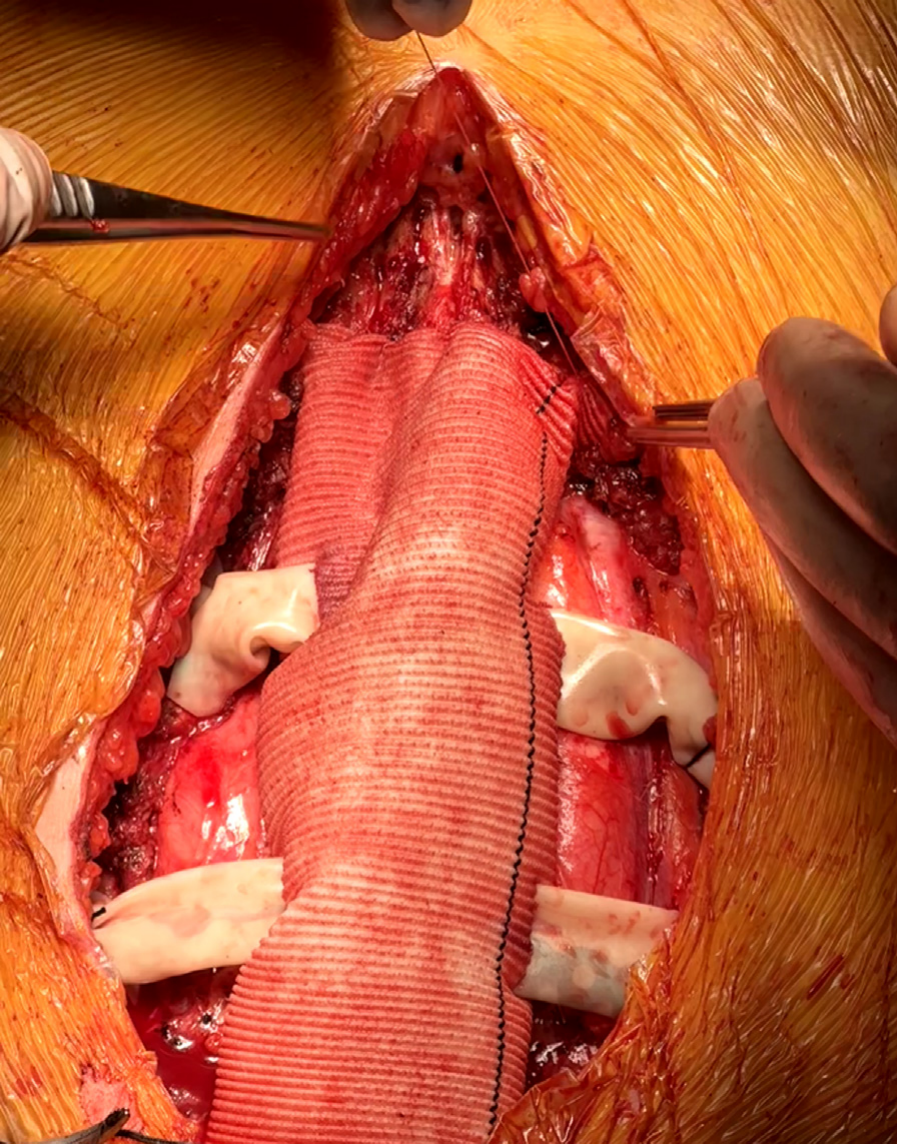
The plates are passed with the latex tube through the Hemashield graft (Getinge AB).

**Figure E6:**
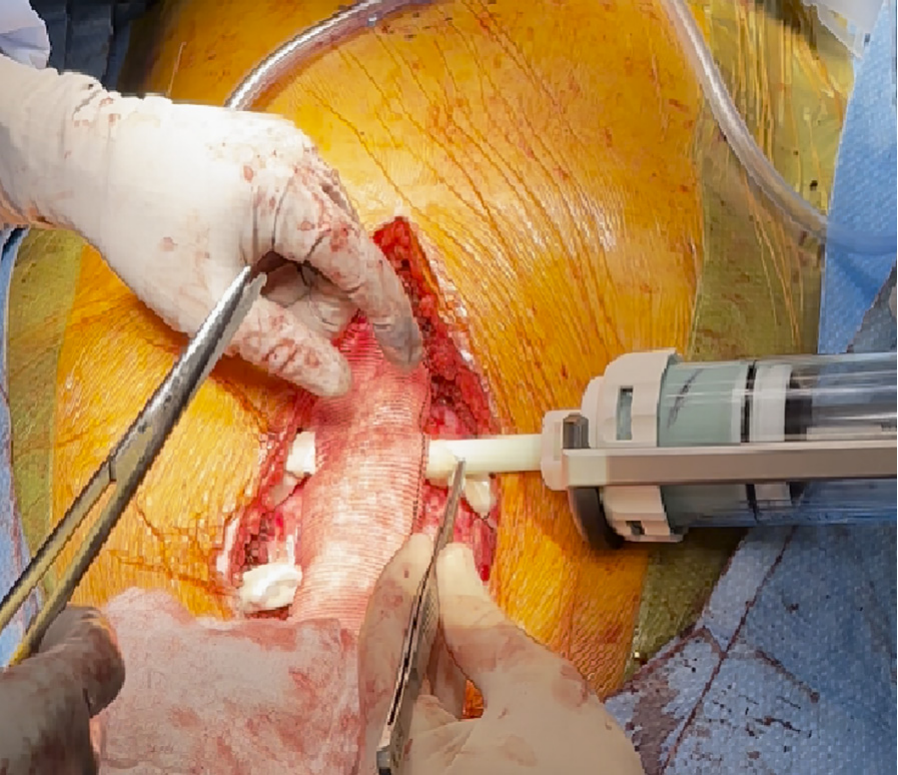
Bone cement is injected into the latex tube to create the neoribs.

**Figure E7:**
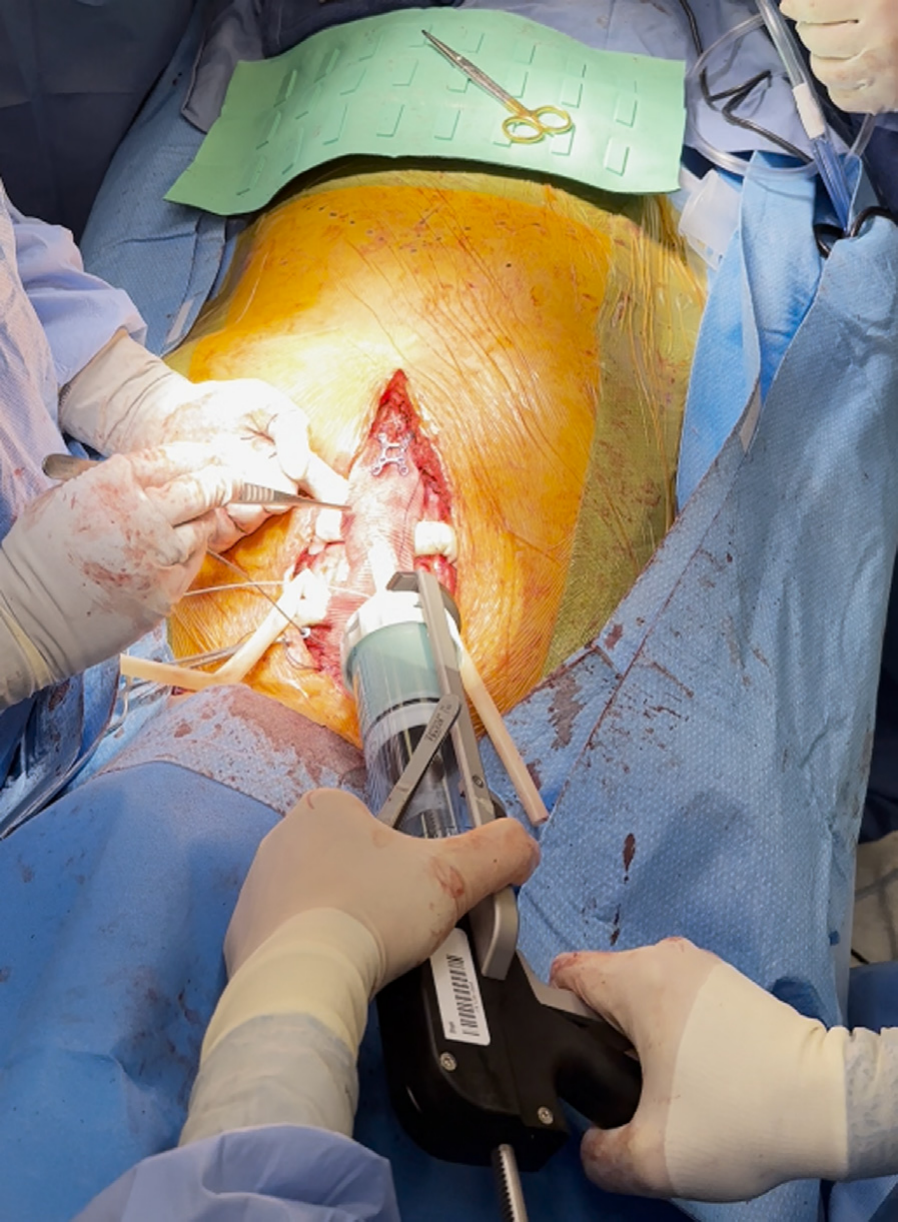
Injection of bone cement into the Hemashield graft (Getinge AB) to cast the sternum.

**Figure E8:**
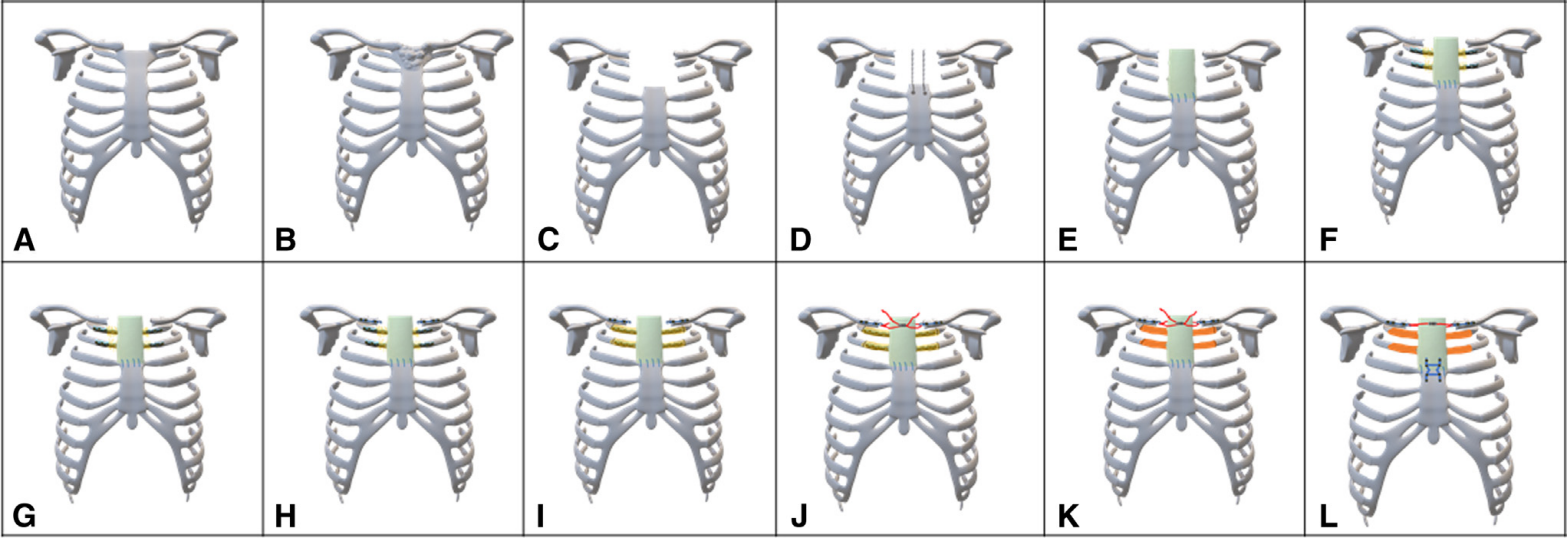
Schematic diagram showing the step-by-step and detailed description of the procedure. A, Normal chest. B, Tumor involving the sternum and the sternoclavicular joint. C, Resection of the tumor with partial sternectomy, resection of medial clavicles, and involved ribs. D, Placement of 2 twisted sternal wires on the sternal stump. E, Placement of the Hemashield graft (Getinge AB) over the wires and affixing it to the sternal stump with 2–4 sutures (Vicryl #0). F, Passing the rib plates (conformed to ribs curvature) with the latex tube crimpled in the middle (to allow placement of the screws), through the Hemashield graft. One plate is used for each level of rib. G, Affixing the plates to corresponding ribs with the screws while the latex tube is held in the midline (for exposure). H, Affixing short plates to clavicle stumps, keeping 1 hole free for the cable. I, The latex tube is pulled over the ribs stumps and temporarily tied with a silk tie (to hold the cement). J, A sternal cable passed through the clavicle plates and through the Hemashield graft and left loose. K, Bone cement is injected into the latex graft (through small hole made for passing the cement device nozzle) to fill them in a volume that matches rib sizes. Once the cement hardens, the latex tube is removed, bone cement is injected into the Hemashield graft (through a small hole made for passing the cement device nozzle) to matching size and volume. Irrigation of the graft as the cement hardens prevents the thermal injury of the graft. L, A plate (*H* or *X* shape) is affixed with Hemashield graft to the sternal stump. Placement of the screws to the Hemashield side should be done before complete hardening of the cement in the graft. The cable holding the clavicles are slightly tightened to become straight with very gentle pull of the clavicles and it is fixated with the crimping device, the excess cable is removed.

At this point, the mold is ready for casting the ribs and the sternum. The latex tube and then the vascular graft are filled with the cement (Figure 1, *C*). As the cement in the graft hardens, a *T*- or an *H*-shaped plate is used to affix the sternal cast to sternal stump. The latex is removed, and the PTFE graft remains in place. The clavicle cable is gently pulled to appropriate tension with light approximation of the clavicular ends and is crimped, allowing hinge motion for the clavicles. The detached sternal ends of the sternocleidomastoid and the pectoralis muscles are sutured to the corresponding anatomic position on the vascular graft and the wound is closed. The surgical steps are described in Figure E1, Figure E2, Figure E3, Figure E4, Figure E5, Figure E6, Figure E7, Figure E8.

## Comments

Multiple techniques have been described for reconstruction after sternectomy.^3^ An optimal reconstruction after sternectomy should preserve the integrity of the bony structure and chest geometry in addition to protection of the mediastinal organs. Whenever soft tissues are resected to achieve clear margins, the reconstruction should include advanced muscle flaps procedures to cover the reconstructed rigid structure. Often, due to complexity of these reconstructions, when the SCJ or the clavicles are resected, the amputated clavicles are not included in the reconstruction; they remain free-floating.

Generally, the reconstruction techniques use native musculocutaneous flaps, soft/rigid patches that provide only defect coverage, and various hardware materials, alone or in combination, to construct a nonanatomic frame that holds the bony structures together. Recently, 3-dimensional-printed patient-customized implants are introduced for reconstructive surgery.^4^ Despite their positive outcomes, their use is limited by requiring extensive preoperative planning and their cost.^5^

The technique described here provides an excellent solution to restore chest wall geometry, stability, and functionality and provides cosmetic results similar to native anatomy (Figure 1, *D*). In this technique, the clavicles are included in the reconstruction and the SCJ function is preserved, adding strength to the structure. This method is easily reproducible and modular and uses readily available supplies and can be used after any type of resection. The mold does not need preoperative planning, and the neosternum cast covered with the PTFE graft provides an optimal base on which the soft tissues can be sutured.

## Conflict of Interest Statement

The author reported no conflicts of interest.

The *Journal* policy requires editors and reviewers to disclose conflicts of interest and to decline handling for which they may have a conflict of interest. The editors and reviewers of this article have no conflicts of interest.
